# Epigenetic Mechanisms Governing Nrf2 Expression and Its Role in Ferroptosis

**DOI:** 10.3390/biomedicines13081913

**Published:** 2025-08-05

**Authors:** Linbo Li, Xinjun Liu, Zizhen Si, Xidi Wang

**Affiliations:** 1 Central Laboratory, The First Affiliated Hospital of Ningbo University, 59 Liuting Street, Haishu District, Ningbo 315010, China; 236002765@nbu.edu.cn (L.L.); 2311140009@nbu.edu.cn (X.L.); 2 Health Science Center, Ningbo University, Ningbo 315211, China; sizizhen@nbu.edu.cn; 3 Ningbo Key Laboratory of Human Microbiology and Precision Medicine, Ningbo 315010, China

**Keywords:** ferroptosis, Nrf2, epigenetics, DNA methylation, histone modification, non-coding RNAs

## Abstract

Ferroptosis is a distinct form of regulated cell death driven by iron-dependent lipid peroxidation participating in various diseases. The nuclear factor erythroid 2-related factor 2 (Nrf2) is a central regulator of cellular redox homeostasis and a key determinant of ferroptosis resistance. Nrf2 activates the expression of downstream antioxidant genes to protect cells from oxidative stress and ferroptosis. Consequently, precise regulation of Nrf2 expression is crucial. Recent studies have revealed that complex epigenetic mechanisms involving DNA methylation, histone modifications, and non-coding RNA networks regulate Nrf2 expression. DNA methylation usually suppresses while histone acetylation promotes Nrf2 expression. The influences of histone methylation on *NFE2L2* are site- and methylation degree-dependent. m6A modification stabilizes *NFE2L2* mRNA to promote Nrf2 expression and thereby inhibit ferroptosis. This article summarizes current understanding of the epigenetic mechanisms controlling Nrf2 expression and Nrf2-mediated ferroptosis pathways and their implications in disease models. The challenges associated with the epigenetic regulation of Nrf2 and future research directions are also discussed. A comprehensive understanding of this regulatory interplay could open new avenues for intervention in ferroptosis-related diseases by fine-tuning cellular redox balance through the epigenetic modulation of Nrf2.

## 1. Overview of Ferroptosis

Cell death is an essential biological process that regulates tissue homeostasis and helps adapt to perturbations in exogenous stimuli and endogenous metabolic states. Historically, based on morphological and biochemical classification criteria, cell death forms have been systematically categorized into two principal categories: programmed cell death (PCD), characterized by regulated signaling cascades, and accidental cell death, marked by uncontrolled cellular collapse. The seminal discovery of developmental apoptosis in *C. elegans* by Brenner, Horvitz [1,2], and Sulston established the conceptual framework for evolutionarily conserved PCD pathways. Subsequent research has identified multiple regulated cell death forms.

**Figure 1:**
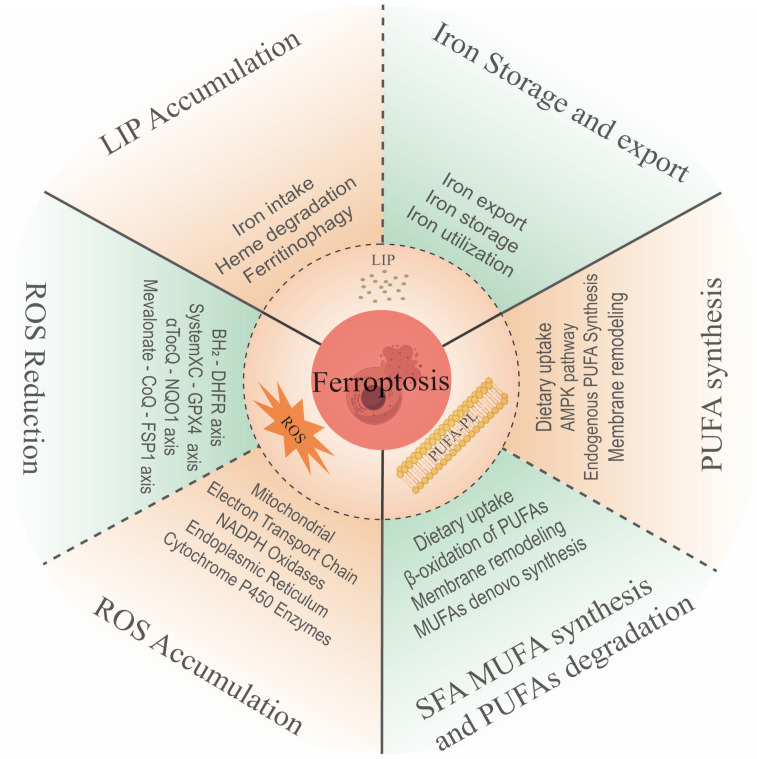
Main pro- and anti-ferroptosis mechanisms. Iron accumulation, ROS accumulation, and PUFA generation are considered main features and mechanisms to drive ferroptosis. Correspondingly, there are different mechanisms to counteract these three mechanisms to inhibit ferroptosis.

Ferroptosis is a regulated cell death modality formally characterized by Dixon et al. in 2012 [3]. It is mechanistically distinct from necrosis, pyroptosis, and autophagy through its iron-dependent lipid peroxidation signature [4,5,6]. This oxidative death process is molecularly defined by systemic collapse of glutathione (GSH)-dependent antioxidant defenses and dysfunctional glutathione peroxidase 4 (GPX4) activity, leading to lethal accumulation of membrane lipid peroxides. The ferroptotic paradigm integrates three core mechanistic axes (Figure 1): (1) metabolic reprogramming of amino acid (cystine-glutamate antiporter system Xc^−^) and lipid pathways; (2) reactive oxygen species (ROS) amplification cascades via Fenton chemistry and lipoxygenase activation; and (3) iron homeostasis dysregulation through transferrin receptor-mediated uptake and ferritinophagy [7].

Current pharmacological ferroptosis inducers (FINs) are classified according to their molecular targets: Class I FINs (e.g., erastin) deplete intracellular GSH pools by inhibiting system Xc^−^ [8,9]; Class II FINs (e.g., RSL3) directly inactivate GPX4 through covalent binding [10]; and Class III FINs (e.g., FIN56) dually suppress GPX4 and ferroptosis suppressor protein 1 (FSP1)/AIFM2 by depleting ubiquinone (CoQ10) precursors via squalene synthase activation [11]. A study using bladder cancer cells (BCs) also indicated that FIN56 works in combination with Torin2 by accelerating degradation of GPX4, yet how this process is achieved requires further investigation [12]; Class IV FINs (e.g., FINO2) promote iron overload through redox-active endoperoxide intermediates [13].

Ferroptosis has emerged as a critical pathophysiological mechanism involved in various human diseases, with its dual roles in disease progression and therapeutic intervention being increasingly elucidated [14]. As an evolutionarily conserved process, ferroptosis plays a crucial role in the development and diseases of various organisms, including plants and animals. Ferroptosis exhibits onco-suppressive effects in oncological contexts by remodeling the tumor microenvironment [15]. For instance, TP53-induced glycolysis and apoptosis regulator (TIGAR) ablation in colorectal cancer models disrupts redox homeostasis (reduced GSH/oxidized glutathione [GSSG] ratio), potentiating ferroptosis sensitivity and significantly attenuating tumor growth [16]. Similar tumor-suppressive outcomes have been documented in acute myeloid leukemia and multiple solid malignancies [17].

The p53 tumor suppressor network is evolutionarily conserved as the “genome guardian” and regulates ferroptosis through non-canonical pathways [18]. Notably, acetylation-deficient p53 mutants retain ferroptosis-inducing ability despite losing classical cell cycle arrest and apoptosis functions. Mechanistically, these mutants transrepress solute carrier family 7 member 11 (SLC7A11), the rate-limiting subunit of system Xc^−^, thereby depleting cysteine (Cys) reservoirs and sensitizing tumors to ferroptotic demise [18,19]. This non-apoptotic tumor suppression paradigm challenges conventional views of p53-mediated oncoprotection, indicating ferroptosis as a therapeutically exploitable vulnerability in p53-mutant cancers [19,20].

In cerebrovascular pathologies, ferroptosis drives ischemic stroke progression via arachidonic acid cascades. During ischemia-reperfusion (I/R) injury, thrombin-mediated phospholipase activation liberates arachidonic acid, which undergoes Acyl-CoA synthetase long-chain family member 4 (ACSL4)-mediated esterification into pro-ferroptotic lipid species. This peroxidation cascade preferentially damages hippocampal and cortical neurons, correlating with cognitive deficits [21,22]. Furthermore, selenocysteine (Sec) biosynthesis defects and developmental brain injuries (periventricular leukomalacia) converge on GPX4 translational failure and GSH system collapse [23].

## 2. The Features of Ferroptosis

### 2.1. Intracellular Iron Accumulation

Iron dyshomeostasis constitutes a biochemical hallmark of ferroptosis, a metabolic vulnerability first mechanistically defined by Stockwell’s group [3]. The labile iron pool (LIP) exerts pro-ferroptotic effects primarily through Fenton chemistry-driven hydroxyl radical generation, which catalyzes non-enzymatic lipid peroxidation cascades (Section 2.3). Numerous main regulatory networks govern ferroptotic sensitivity through iron metabolism. Heme degradation-derived iron released via heme oxygenase-1 (HO-1) activity directly fuels lipid ROS production. HO-1 induction correlates with ferroptosis susceptibility across multiple models (Chapter 2) [24,25]. Ferritinophagy, an NCOA4-mediated selective autophagic degradation of ferritin, releases stored intracellular iron and increases the expression of transferrin receptor 1 [26,27], which causes iron accumulation and subsequently enhanced enzymatic and non-enzymatic lipid peroxidation reactions induced by free iron.

### 2.2. Intracellular ROS Accumulation

ROS are intrinsically produced through fundamental cellular processes such as oxidative phosphorylation, lipid remodeling, and amino acid catabolism, serving as critical mediators of ferroptotic lipid peroxidation [28,29]. The peroxidation cascade is mainly driven by hydroxyl radicals (OH) produced via iron-catalyzed Haber–Weiss reactions, which oxidize polyunsaturated fatty acid (PUFA)-enriched phospholipids within cellular membranes. Mitochondria comprise the primary cellular source of pro-ferroptosis ROS due to their unique biochemical properties: (1) high OH yield from electron transport chain (ETC) leakage during oxidative phosphorylation, (2) PUFA-dense inner membrane architecture prone to peroxidation, and (3) iron-sulfur cluster biogenesis maintaining redox-active iron pools. Cells contain numerous mitochondrial-targeted ROS scavengers to counteract ROS produced by electron leakage in the ETC. In addition to the previously reported apoptosis-inducing factor [30], the knockdown of MitoQ and BID, as well as the recently reported TP53-regulated fructose-2,6-bisphosphatase-like activity protein TIGAR, have all been demonstrated to significantly eliminate mitochondrial ROS [31,32,33]. The E3 ubiquitin ligase RBK1 can significantly promote the K48 ubiquitination of MFN2, a key mitochondrial outer membrane protein, thereby facilitating its proteasomal degradation [34]. This reduces mitochondrial fusion and lowers ROS production, which is critical under cellular stress conditions [35]. Furthermore, ROS damages mitochondria to reduce NAD(P)H generation and subsequently disrupts the pentose phosphate pathway (PPP) [36]. Exogenous FINs, such as erastin, inhibit cystine transport, bind to mitochondrial voltage-dependent anion channel (VDAC), and alter its conformation [37]. This reopens the mitochondrial outer membrane channel, reactivating the tricarboxylic acid cycle in cancer cells. Additionally, erastin treatment can induce the ubiquitination and degradation of VDAC, producing mitochondrial ROS [38].

### 2.3. Lipid Peroxidation as a Key Process of Ferroptosis

Lipid peroxidation in ferroptosis progresses through two interconnected axes: ROS amplification and membrane lipid remodeling. This process involves enzymatic drivers (lipoxygenases [LOXs] and peroxidoreductases) and non-enzymatic Fenton reactions, as previously described [14,33]. Notably, redox-active iron catalyzes the conversion of lipid hydroperoxides (PLOOH) to alkoxyl radicals (L-O), initiating chain reactions that oxidize PUFA-containing lipids (LH) into (L) [39]. These propagating radicals establish self-amplifying peroxidation loops, ultimately triggering ferroptosis upon membrane lipid hydroperoxide overload.

Lipid peroxidation mainly initiates within the endoplasmic reticulum (ER) membrane, subsequently propagating to the plasma membrane and other organelles, with mitochondria representing the most prominent example [40]. ER and Golgi membranes serve as critical platforms for ferroptosis induction. Although there is no need for mitochondrial membrane peroxidation for ferroptosis execution, it can dynamically regulate cellular susceptibility to ferroptosis under specific pathophysiological conditions [41].

PUFAs, characterized by multiple oxidation-prone bis-allylic bonds, are central lipid peroxidation mediators. PUFAs are categorized into two major classes: omega-3 and -6 fatty acids, which have been implicated in various pathological conditions, including neurodevelopmental disorders such as ADHD [42,43]. PUFAs exhibit tumor-suppressive effects through ferroptosis activation in malignant cells [44].

The ferroptosis process triggered by RSL-3 involves GSH depletion and subsequent peroxidation of polyunsaturated fatty acid-containing phospholipids (PUFA-PLs), mediated by LOXs such as ALOX. RSL-3 specifically acts as a covalent inhibitor of GPX4 by targeting its Sec and Cys residues [45]. While free PUFAs generally remain non-toxic to cells, their conjugation to glycerophospholipids via ACSL4 significantly enhances membrane PUFA-PL levels [46], promoting ferroptotic susceptibility. Recent investigations reveal that interferon-γ (IFNγ) derived from CD8^+^ T cells upregulates *ACSL4* expression through IFN receptor (IFNR)-mediated signaling and the transcription factor (TF) IRF1, thereby accelerating the lipid peroxidation cascade [47]. Conversely, carnitine palmitoyltransferase 1A (CPT1A), a mitochondrial fatty acid transporter, has been demonstrated to suppress *ACSL4* expression and protect cancer stem cells from ferroptosis by facilitating fatty acid β-oxidation [48]. Two major lipid classes demonstrate susceptibility to oxidative modification during ferroptosis: arachidonic acid-containing and eicosapentaenoic acid phospholipids [49,50]. The enzymatic processing of unsaturated fatty acids plays dual regulatory roles. While cyclooxygenase-2 is a positive regulator of ferroptosis through prostaglandin synthesis [51,52,53], lipid peroxidation via ALOX-mediated oxidation of PUFA-PLs represents another crucial molecular pathway driving ferroptosis.

In contrast to their polyunsaturated counterparts, monounsaturated fatty acid-containing phospholipids exhibit ferroptosis-suppressive properties by competitive displacement of PUFA-PLs from cell membranes [54]. This protective effect is amplified by ACSL3, which catalyzes the esterification of free MUFAs into membrane phospholipids, thereby conferring cellular protection against ferroptotic damage [55]. The lipid peroxidation process in ferroptosis demonstrates remarkable regulatory complexity, involving dynamic equilibrium between pro-oxidative drivers and antioxidant defense systems. Targeting these particular systems may provide therapeutic opportunities for treating related diseases.

## 3. Intracellular Ferroptosis Resistance System

### 3.1. GPX4-GSH System and Membrane Lipid Transport System Resist Ferroptosis

GPX4 is a critical lipid hydroperoxidase through its redox cycling mechanism [56]. This selenoprotein catalyzes the reduction of lipid peroxides (PUFA-OOH) to corresponding alcohols (L-OH) by utilizing GSH as an electron donor. The catalytic tetramer structure, comprising Sec and tryptophan, glutamine, and asparagine residues, enables its antioxidant activity. The selenol group (GPX4-Se-H) at the active site initiates reduction by donating electrons to lipid peroxides, transforming into an oxidized selenenic acid intermediate (GPX4-Se-OH). Subsequent conjugation with GSH generates a selenylsulfide adduct (GPX4-Se-SG) and a water molecule. Regeneration of the reduced enzyme state occurs through a second GSH-mediated reduction, producing GSSG [57]. This GSH-dependent catalytic cycle underpins the essential role of GPX4 in ferroptosis prevention. Pharmacological agents such as buthionine sulfoximine exploit this pathway by blocking γ-glutamylcysteine synthetase, thereby depleting GSH and inducing ferroptosis [58]. The regulatory network extends to nuclear factor erythroid 2-related factor 2 (Nrf2), whose phosphorylation at Ser40 facilitates nuclear translocation and transcriptional activation of genes involved in GSH biosynthesis and antioxidant defense [59,60]. Carnitine CPT1A modulates this axis through a CPT1A/c-Myc feedback loop that enhances Nrf2 activity [48].

FINs exhibit multimodal mechanisms targeting GPX4 functionality. While copper promotes GPX4 ubiquitination via Cys residue modification [61], classical inducer RSL3 was recently demonstrated to primarily inhibit thioredoxin reductase 1 (TXNRD1) rather than directly suppressing GPX4 activity [62]. This discovery challenges conventional understanding, as TXNRD1 regulates thioredoxin (Trx)-mediated cystine reduction and GPX4 biosynthesis [63]. Supporting evidence comes from studies demonstrating that nicotinamide riboside (NR) supplementation enhances hepatic nicotinamide adenine dinucleotide phosphate (NADPH) levels and *TXNRD1* expression, effectively mitigating lipid peroxidation [64]. Although auranofin and other thioredoxin reductase inhibitors (TRi) compounds exhibit ferroptosis-inducing potential through TXNRD1 inhibition [65], their clinical applicability and precise mechanistic relationships require systematic investigation.

The cystine/glutamate antiporter System Xc^−^, a heterodimeric membrane transport complex comprising SLC7A11 and SLC3A2 subunits, operates synergistically with the GPX4-GSH antioxidant axis to maintain redox homeostasis [66]. This exchange system mediates the 1:1 counter transport of extracellular cystine and intracellular glutamate, providing essential Cys precursors for GSH biosynthesis [63,67,68,69]. Pharmacological inhibitors of System Xc^−^, including first-generation FINs such as erastin, exert their effects primarily through competitive binding to the SLC7A11 subunit, thereby compromising cystine uptake and subsequent GSH biosynthesis. Clinical compounds with ferroptosis-inducing properties, notably sorafenib and sulfasalazine, share this mechanism by targeting System Xc^−^ functionality [15]. Transcriptional regulation of SLC7A11 involves a complex network of epigenetic modulators: The BRCA1-associated protein BAP1 and TF ATF3 function as transcriptional repressors, while Nrf2 and ATF4 act as positive regulators [66,70]. Of particular therapeutic relevance, the tumor suppressor p53 enhances ferroptosis susceptibility through dual mechanisms—directly suppressing *SLC7A11* expression and allosterically inhibiting System Xc^−^ transport activity, ultimately depleting cellular antioxidant reserves and precipitating lipid peroxidation-mediated cell death [71].

### 3.2. NADPH System in Ferroptosis Resistance

The NADPH system acts as the principal cellular redox buffer, sustaining reducing equivalents required for fundamental biological processes, including antioxidant defense [72], metabolic regulation [73], and biosynthetic pathways [74]. NADPH production mainly occurs through the PPP [73,75]. This coenzyme functions as an essential electron donor for two critical antioxidant systems: (1) The Trx system, where NADPH reduces oxidized thioredoxin (Trx-S2) to its active dithiol form (Trx-(SH)2) [76], and (2) glutathione reductase, which regenerates reduced GSH for lipid peroxide detoxification [77,78]. While malignant cells maintain elevated NADPH levels to support lipogenesis and oxidative stress mitigation, emerging evidence reveals the paradoxical roles of NADPH in pro-oxidant systems. Specifically, NADPH provides reducing equivalents to cytochrome P450 (CYP450) oxidoreductase, energizing CYP450-mediated oxidation reactions that interestingly enhance cellular oxidative burden [79]. Genetic ablation of CYP450 isoforms significantly diminishes ferroptosis susceptibility across various malignancies, including ovarian, endometrial, hepatic, colorectal, pulmonary, and pancreatic carcinomas [80,81]. However, the canonical antioxidant function of NADPH, particularly its capacity to reduce lipid peroxides, remains its primary mechanism for counteracting ferroptotic cell death.

FSP1/AIFM2 is an NADPH-dependent oxidoreductase that confers ferroptosis resistance through GPX4-independent antioxidant mechanisms [82,83]. FSP1 utilizes NADPH as a coenzyme to reduce CoQ10 to ubiquinol, a powerful reducing agent that reduces peroxidized PUFAs [84]. FSP1 is localized to the cell membrane, which allows it to act straightforwardly.

## 4. Overview of Nrf2

Nrf2, a cap’n’collar-basic leucine zipper (bZIP) TF [85], regulates cellular defenses against oxidative stress through its seven conserved Neh domains. The Neh2 domain mediates Keap1-dependent proteasomal degradation [86], while the serine-rich Neh6 domain facilitates Keap1-independent regulatory mechanisms [87]. Structural functionality arises from the Neh1 domain’s bZIP motif, enabling heterodimerization with small Maf (sMaf) proteins for DNA binding [88]. Ubiquitously expressed across human cell types, Nrf2 maintains redox homeostasis while influencing cellular proliferation and differentiation pathways [89,90,91].

The Nrf2/antioxidant response element (ARE) axis activation occurs through oxidative stress-induced conformational changes in Keap1. Mechanistically, hydrogen peroxide-mediated ER stress triggers Ca^2+^ efflux, potentiating mitochondrial H_2_O_2_ generation. This redox imbalance induces Cys151-dependent disulfide bond formation within Keap1 homodimers, disrupting Nrf2 ubiquitination and enabling the nuclear accumulation of stabilized Nrf2 [88].

### 4.1. Role of Nrf2 in Oxidative Stress

Nrf2 coordinates cellular responses to oxidative stress through transcriptional regulation of antioxidant genes, effectively reducing ROS production. This redox regulator maintains oxidative mediator equilibrium, preserving redox homeostasis by activating ARE-dependent gene transcription [92]. As detailed previously, Nrf2 upregulates antioxidant genes, bolstering cellular antioxidant defenses. The canonical Nrf2/HO-1 axis inhibits ROS and malondialdehyde (MDA) production while suppressing NADPH oxidase activity, concurrently enhancing superoxide dismutase (SOD) and glutathione peroxidase (GSH-Px) activation [93,94]. Nrf2 further modulates mitochondrial function and metabolic pathways to attenuate ROS production.

Beyond ferroptosis regulation, Nrf2-activated genes participate in DNA repair mechanisms [95], inflammatory resolution pathways [96], and apoptosis inhibition, collectively promoting cellular survival. Clinical evidence from type II papillary renal cell carcinoma studies has identified hyperactivation of the Nrf2/ARE pathway as a molecular hallmark, particularly in tumors exhibiting the CpG island methylator phenotype. Somatic mutations in *NFE2L2* (encoding Nrf2), *CUL3*, and *Keap1* drive constitutive Nrf2 activation, with resultant overexpression of oxidative stress-responsive genes such as NADPH quinone dehydrogenase 1 (*NQO1*) correlating with poor clinical outcomes and reduced survival rates [97]. In non-small cell lung cancer (NSCLC), acquired Nrf2 hotspot mutations demonstrate functional synergy with secondary anaplastic lymphoma kinase (ALK) mutations, conferring therapeutic resistance to second-generation ALK inhibitors [98].

Nrf2 responds to oxidative stress by regulating the expression of multiple antioxidant enzyme genes. NQO1 (NADPH: quinone oxidoreductase 1) protects cells from oxidative damage by reducing the toxicity of quinone compounds [99,100]. Additionally, Nrf2 regulates key components of the endogenous antioxidant system, particularly in the synthesis of GSH. It does this by controlling the expression of the transport system subunit xCT and the expression of the two key enzymes involved in GSH synthesis: the glutamate-Cys ligase catalytic (GCLC) and the modifier subunit (GCLM). SOD catalyzes the dismutation of superoxide anion radicals into hydrogen peroxide and oxygen, thereby reducing the accumulation of superoxide free radicals [101,102,103]. Studies have indicated that SOD functions in cytoplasm and also plays a role in the clearance of mitochondrial superoxide anions [104,105]. GPX, as previously mentioned, is a key enzyme in reducing lipid peroxide and preventing oxidative damage to cell membranes. In a diabetic nephropathy model using Akita mice, Nrf2-knockout mice exhibited downregulation of genes related to GSH synthesis, enhanced mesangial dissolution in the distal tubules, and severe inflammation and elevated macrophage levels. Conversely, these symptoms were suppressed in *Keap1*-knockdown mice [106]. In hepatocellular carcinoma (HCC), Nrf2 deficiency significantly reduced histone acetylation levels, particularly H3K27 acetylation, which affected the assembly of transcription complexes and resulted in reduced expression of various downstream antioxidants and metabolic genes [107]. This suggests the antioxidant role of Nrf2 in promoting tumor progression. Overall, Nrf2 is crucial in maintaining cellular homeostasis in response to intracellular ROS and electrophiles.

### 4.2. Role of Nrf2 in Ferroptosis

Extensive research has established the critical role of ferroptosis in tumor biology. Nrf2 exerts multifaceted anti-ferroptotic effects through coordinated regulation of lipid peroxidation defense mechanisms. Its transcriptional targets in ferroptosis prevention span three key metabolic axes: iron homeostasis [108], intermediary metabolism, and GSH biosynthesis [109]. Notably, Nrf2 regulates the ubiquitin ligase FBXL5 and the E3 ubiquitin ligase HERC2, the latter functioning as a co-activator for NCOA4 [110]. Genetic ablation of Nrf2 stabilizes FBXL5, which enhances ubiquitination-mediated degradation of iron regulatory protein 2, thereby promoting iron-binding protein synthesis. Concurrent stabilization of NCOA4 facilitates autophagosomal recruitment of iron-binding proteins [26,111], finally leading to the accumulation of apoferritin/NCOA4, increasing LIP. Furthermore, Nrf2 deficiency disrupts autophagosome–lysosome fusion via the mTOR-TFEB-E-box signaling axis by indirectly suppressing SNARE family protein VAMP8 expression [111]. These coordinated molecular alterations collectively destabilize iron-binding protein equilibrium, culminating in LIP accumulation.

The Nrf2 signaling pathway becomes transcriptionally activated under ferroptosis-inducing stress conditions. The challenge of oxygen–glucose deprivation/reperfusion (OGD/R) induces Nrf2 upregulation, subsequently elevating *SLC7A11* expression to confer ferroptosis resistance [112]. In erastin- or doxorubicin (DOX)-treated cardiomyocytes, coordinated upregulation of prostaglandin E2 and its EP1 receptor activates the PKC/Nrf2 axis to transcriptionally upregulate GPX4 and SLC7A11, thereby counteracting DOX-induced ferroptotic cell death [113]. Moreover, exposure to bisphenol A (BPA) in pregnant CD-1 mice stimulates *Nrf2* expression and its direct binding to the *Srebp-1c* promoter. This mechanism drives the accumulation of lipids in the liver, as demonstrated in studies [114]. Notably, multiple phytochemicals exhibit therapeutic potential through Nrf2-mediated ferroptosis inhibition. Plant-derived compounds, including quercetin, sulforaphane, resveratrol, curcumin, luteolin, corosolic acid, and apigenin, demonstrate Nrf2-activating ability across experimental models, thereby effectively suppressing ferroptosis cellular systems and animal studies. The combination of these agents, particularly with curcumin co-treatment, results in synergistic anti-ferroptotic effects [115,116,117,118,119]. Consequently, Nrf2 regulators maintain cellular homeostasis by coordinately optimizing iron handling, intermediary metabolic flux, and GSH biosynthesis to effectively prevent ferroptosis [52].

Nrf2 expression undergoes significant upregulation under ferroptosis-inducing conditions, such as RSL3 stimulation. By inducing Nrf2 protein expression, RSL3 suppresses inflammatory cytokine transcription through inhibition of RNA polymerase II recruitment to pro-inflammatory gene promoters, thereby protecting cells from ferroptosis [112]. Nrf2-mediated regulation of iron metabolism-related genes mitigates intracellular free iron accumulation, consequently attenuating iron-catalyzed lipid peroxidation. The antioxidant genes *HO-1* and *GPX4*, transcriptionally activated by Nrf2, effectively reduce lipid peroxidation, though this protective axis is counteracted by cetuximab treatment [120]. Nrf2 further upregulates heme synthesis enzymes, including ferrochelatase (FECH) and ATP-binding cassette subfamily B member 6 (ABCB6), reducing LIP accumulation. Functional coordination between FSP1 and Nrf2 appears facilitated by NADPH production. During exogenous stress responses, Nrf2 enhances NADPH regeneration via β-oxidation and glucose metabolism reprogramming, thereby regulating biomacromolecule degradation and optimizing intermediary metabolism [121]. Moreover, Nrf2 governs GSH biosynthesis through transcriptional activation of GCLC and GCLM, suppressing lipid peroxidation-driven ferroptosis. Under Cys-depleted conditions, GCLC catalyzes γ-glutamylcysteine synthesis from glutamate, preventing ferroptosis induced by glutamate overload [122].

Certain exogenous therapeutics exhibit their pro-ferroptotic effects via Nrf2 pathway modulation, as demonstrated by curcumin [123,124]. In human osteosarcoma models, curcumin sensitizes malignant cells to ferroptosis by downregulating Nrf2/GPX4 axis components. Therapeutically, the ferroptosis inhibitor Liproxstatin-1 and the Nrf2 activator bardoxolone methyl effectively counteract curcumin-induced cytotoxicity, confirming the key regulatory role of Nrf2 [125].

Beyond its canonical transcriptional regulation of antioxidant genes, Nrf2 regulates ferroptosis-associated pathways through epigenetic mechanisms. Specifically, Nrf2 modulates the expression of *RANKL*, a critical factor in osteoclast formation influenced by osteocyte-mediated ferroptosis. This regulation occurs via the methylation of the *RANKL* promoter by DNA methyltransferase 3A (DNMT3A) [126]. Environmental toxicant exposure studies reveal the capacity of Nrf2 to regulate CYP450 enzymes through promoter-specific epigenetic modifications. During PM2.5 exposures, Nrf2 establishes distinct methylation patterns in *CYP2E1*, *CYP1A1*, and *CYP2S1* promoters. Notably, CYP2E1, a multifunctional monooxygenase involved in xenobiotic metabolism, toxin clearance, and lipid oxidation, undergoes Nrf2-dependent hypermethylation at CpG islands, attenuating CYP2E1 activity and associated ferroptosis risk [127,128]. Some studies indicate that environmental toxicants can inhibit the nuclear translocation of Nrf2 [129].

The Nrf2-TXNRD1 axis comprises a pivotal regulatory mechanism conferring ferroptosis resistance [130]. Nrf2 transcriptionally activates *TXNRD1* with the iron chelator deferoxamine (DFO), enhancing Nrf2 nuclear translocation in subarachnoid hemorrhage mouse models [92]. This DFO-induced Nrf2/TXNRD1 axis activation alleviates neuronal ferroptosis. Furthermore, under Nrf2 regulation, HO-1 catalyzes heme degradation to produce the antioxidant bilirubin while simultaneously releasing LIP components [131]. Nrf2 also coordinates heme biosynthesis via transcriptional control of *FECH*, mediating iron incorporation into protoporphyrin IX and ABCB6, which facilitates mitochondrial import of coproporphyrinogen III. By enhancing heme synthesis, Nrf2 significantly reduces intracellular LIP accumulation and attenuates oxidative stress [108,132].

## 5. Epigenetic Regulatory Mechanisms of Nrf2 in Ferroptosis

**Figure 2:**
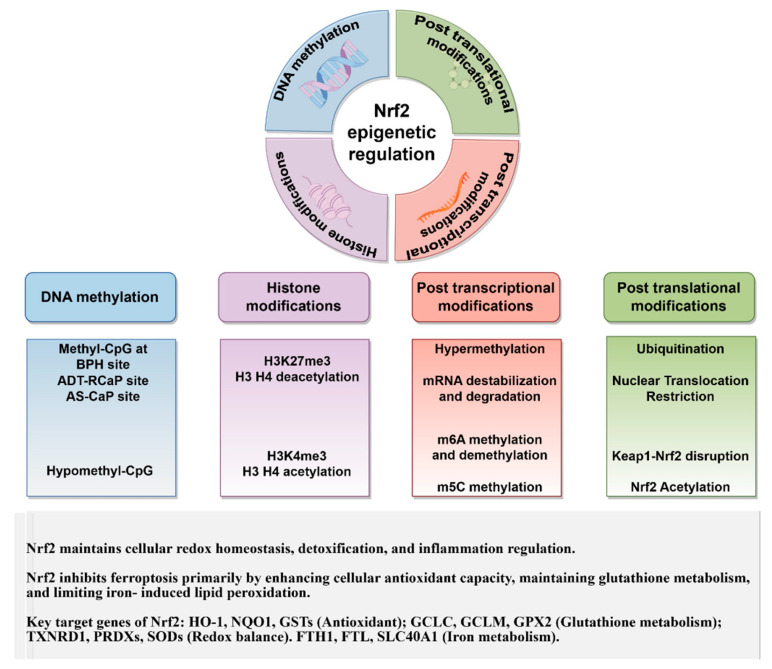
The epigenetic mechanisms govern Nrf2 expression and their roles against ferroptosis.

Epigenetic modifications involve heritable changes in phenotype that occur without alterations to the DNA sequence. These modifications include DNA methylation, histone acetylation, regulation by long non-coding RNAs, miRNA interactions, and mRNA N^6^-methyladenosine (m^6^A) modifications. At the core of these processes are covalent histone modifications and non-covalent chromatin remodeling events that collectively modulate chromatin architecture and functional states [133]. Recent evidence indicates the significance of epigenetic regulatory networks in determining the dynamics of Nrf2 expression (Figure 2). Importantly, interaction among different epigenetic mechanisms has been documented. For instance, histone H3 lysine 36 trimethylation facilitates DNMT recruitment through Pro-Trp-Trp-Pro domain interactions, establishing DNA methylation patterns. In contrast, H3K4 methylation inhibits the enzymatic activity of DNMT3 [134].

Recent studies have revealed that nucleotide-level epigenetic changes can occur through base conversion mechanisms. The activation-induced cytidine deaminase/apolipoprotein B mRNA-editing enzyme complex deaminates 5-hydroxymethylcytosine (5hmC) and converts it into 5-hydroxymethyluracil, illustrating such base-type modifications [135]. However, the functional implications of Nrf2-associated epigenetic modifications in ferroptosis pathophysiology remain incompletely characterized, warranting systematic investigation.

### 5.1. Impact of DNA Methylation on Nrf2 in Ferroptosis

In mammalian systems, DNA methylation primarily appears as 5-methylcytosine (5mC) at cytosine residues within CpG dinucleotides, historically referred to as the “fifth base” of DNA [136]. Transcriptional silencing occurs when promoter CpG island methylation spatially hinders TF recognition, effectively repressing gene expression [137]. This epigenetic modification is regulated by three enzymatically active DNMT3A, including DNMT1, DNMT3A, and DNMT3B. These enzymes catalyze the transfer of methyl groups from S-adenosylmethionine (SAM) to the C5 positions of cytosine. Collectively, these α-ketoglutarate (α-KG)-dependent enzymes maintain the fidelity of methylation during DNA replication [138]. Passive DNA demethylation can occur through the dilution of methylation marks during replication when DNMTs are inhibited. Experimental evidence from TPA-induced JB6 P+ mouse epidermal cells demonstrates that delphinidin mediates the suppression of DNMT1 and DNMT3A via hypomethylation at the *NFE2L2* promoter’s CpG sites, which activates the Nrf2-ARE pathway. Additionally, complementary studies in TRAMP-C1 prostate cancer models confirm *NFE2L2* reactivation using DNMT inhibitor 5-aza-2′-deoxycytidine and histone deacetylase inhibitor Trichostatin A [139,140].

Active DNA demethylation involves ten-eleven translocation (TET) dioxygenases (TET1/2/3) that iteratively oxidize 5mC to 5hmC, 5-formylcytosine (5fC), and 5-carboxylcytosine (5caC) [135,136,141]. Thymine DNA glycosylase excises 5fC/5caC, allowing base excision repair-mediated restoration. In 5-fluorouracil-resistant colon cancer cells, ROS-driven TET1 upregulation couples with Nrf2-mediated HO-1 induction to confer ferroptosis resistance [142]. Erythroid systems under iron dysregulation reveal that TET2-dependent *NFE2L2* hypomethylation activates FPN and erythroferrone expression, restoring iron homeostasis [143]. Clinical analyses of 27 prostate cancer specimens and LNCaP cells via MAQMA and bisulfite sequencing identified three hypermethylated *NFE2L2* promoter CpG sites that suppress transcriptional activity [139]. These findings underscore *NFE2L2* promoter methylation status as a key determinant of ferroptosis susceptibility, suggesting potential strategic targets for therapeutic intervention [144].

As a key regulator of oxidative stress responses, the methylation status of the *NFE2L2* plays a crucial role in the pathophysiology of ferroptosis. Controlled physical activity modulates DNA methylation patterns, with recent evidence suggesting that exercise-induced epigenetic changes in NFE2L2 may help reduce inflammation [145,146], prevent carcinogenesis, and mitigate neurodegeneration [147]. Notably, treadmill running in ovariectomized murine models restored physiological DNMT activity and reversed *NFE2L2* promoter CpG island hypermethylation in osteoblasts, demonstrating exercise-mediated epigenetic normalization [148,149].

Pathological hypermethylation at two conserved *NFE2L2* promoter CpG sites has been observed in cells derived from patients with chronic obstructive pulmonary disease and cigarette smoke extract-treated models. This aberrant methylation epigenetically silences *NFE2L2*, suppressing the Nrf2/GPX4 antioxidant axis while elevating ROS and MDA levels [150]. Furthermore, clinical analyses revealed significantly elevated *NFE2L2* methylation in preeclamptic placental tissues, correlating with reduced antioxidant capacity and elevated systemic oxidative stress markers [151]. Interestingly, these chronic oxidative adaptations may enhance cellular tolerance to ferroptotic stimuli, indicating that *NFE2L2* methylation may play context-dependent roles in maintaining redox homeostasis.

Chronic iron exposure induces significant genome-wide hypomethylation in colonic epithelial cells. In vitro treatment of murine intestinal epithelial cells with 10 µM iron triggered hypomethylation at *NFE2L2* promoter CpG islands, with more pronounced hypomethylation observed in downstream Nrf2 pathway targets such as GPX2 and NQO1 [152]. Seminal investigations have demonstrated that the reversal of CpG promoter methylation status reactivates *NFE2L2* transcription, restoring the expression of its downstream antioxidant effector genes, including *NQO1* [153]. Mechanistic studies reveal that intracellular LIP accumulation and concomitant lipid peroxidation are critical determinants of this hypomethylation cascade. This indicates that cells adapt to iron-rich environments by coordinating the hypomethylation of the *NFE2L2* promoter and its transcriptional targets to mitigate ferroptotic stress [154]. Overall, these findings establish *NFE2L2* promoter methylation as a key regulator of cellular redox sensitivity. However, hypermethylation can compromise the networks of oxidative stress response genes, thereby increasing susceptibility to ferroptosis.

### 5.2. Impact of Histone Modifications on NFE2L2 Expression and Function on Ferroptosis

The discovery of histone post-translational modifications (PTMs) in 1964 marked a significant advance in the field of epigenetics. Initial observations suggested that these modifications play a regulatory role in RNA synthesis [155]. The concept was further refined through the histone code hypothesis, which proposed that combinatorial patterns of these modifications—such as acetylation, methylation, phosphorylation, and ubiquitination—determine the structural states of chromatin and the resulting transcriptional outputs. This mechanism allows for complex cellular signaling networks and gene regulatory plasticity [156]. Contemporary research has indicated that these chemical alterations dynamically modulate nucleosome conformation, influencing chromatin accessibility and transcriptional competence. This regulation is achieved through the coordinated recruitment of chromatin remodelers and TFs.

#### 5.2.1. Histone Acetylation Modification

Histone acetylation is a crucial epigenetic mechanism in transcriptional regulation. It primarily promotes gene activation by facilitating the recruitment of trans-acting factors to chromatin [157]. This modification mainly affects lysine residues on histones H3 (K9, K14, K18, and K23) and H4. The site-specific acetylation neutralizes the positive charge of lysine, which weakens the interactions between histones and DNA. The resultant chromatin relaxation enhances accessibility for the transcriptional machinery. The process is dynamically regulated by the opposing actions of histone acetyltransferases (HATs) and histone deacetylases (HDACs) [156]. Pathological alterations in histone acetylation patterns are observed across malignancies, inflammatory disorders, oxidative stress conditions, and metabolic syndromes. These alterations often correlate with the dysregulation of different HDAC isoenzymes [158].

Recent evidence underscores the significant role of histone acetylation in regulating ferroptosis through the modulation of *NFE2L2*. Clinical analyses reveal HDAC4 overexpression in patients with diabetic foot ulcers inversely correlates with *NFE2L2* expression [159], suggesting that deacetylation may suppress *NFE2L2* activity. Preclinical research indicates that HDAC inhibitors, such as trichostatin A, enhance the acetylation of the *NFE2L2* promoter in murine models of osteoarthritis, thereby restoring Nrf2-driven antioxidant defenses [160,161]. UHRF1-dependent acetylation at the Keap1 locus further implicates epigenetic crosstalk in Nrf2 regulation. Notably, pterostilbene mitigates oxidative stress-induced ferroptosis in COV434 ovarian granulosa cells by boosting the activity of the Nrf2/HO-1 pathway through acetylation-dependent transcriptional activation [162]. Pharmacological interventions targeting HDAC1, such as 7-hydroxycoumarin, counteract polymyxin B-induced renal oxidative damage by restoring *NFE2L2* acetylation status, indicating their therapeutic potential [163].

A secondary mechanism involves acetylation-dependent modulation of Nrf2 chromatin localization. In models of hepatic inflammation induced by a high-salt diet, hyperacetylation of the SIRT3 promoter impairs Nrf2 binding and worsens pro-inflammatory cytokine production [164]. In NSCLC, GAS41 recognizes H3K27 acetylation marks at the SLC7A11 promoter, stabilizing Nrf2-chromatin interactions to enhance antioxidant gene transcription. GAS41 knockout enhances lipid peroxidation and reduces tumor burden by 75% via ferroptosis induction, suggesting the pathophysiological relevance of this axis [165].

Histone modification is also involved in regulating *NFE2L2* expression during aging [160]. Histone deacetylation and repressive methylation reduce *NFE2L2* transcription by closing chromatin. HDACs and SIRT1 are known to interact with KEAP1/NRF2 signaling, indirectly affecting its nuclear translocation and DNA binding [166]. Jawad et al. showed that supplementing porcine embryos with myo-inositol upregulates NRF2 activity via KEAP1/NRF2 signaling, restoring mitochondrial function and reducing apoptosis, suggesting an epigenetically responsive NRF2 axis [167].

#### 5.2.2. Histone Methylation Modifications

Histone lysine and arginine residues undergo mono-, di-, or tri-methylation through the enzymatic activity of histone methyltransferases. The reversibility of these modifications is regulated by histone demethylases [168]. The transcriptional consequences of histone methylation are site-dependent, with distinct functional roles emerging from differential spatial enrichment. Methylation marks such as H3K79, H3K4, H3K27, H4K20, and H2BK5 are primarily located near transcription start sites [169,170], while H3K36 methylation clusters near transcription termination sites [171]. Functionally, H3K4 and H3K79 methylation demonstrate transcriptional activation properties. In 5-fluorouracil-resistant colon cancer models, upregulation of the histone methyltransferase MLL and its trimethylation product H3K4me3 correlates with enhanced *NFE2L2* expression [172]. Conversely, Polycomb repressive complex 2 epigenetically silences *NFE2L2* via H3K27me3 deposition in lung cancer, exerting tumor-suppressive effects [171,173].

Dietary factors significantly influence this regulatory axis. For instance, a high-sugar intake promotes methylation at the GCLC-ARE4 element, impairing Nrf2 binding and subsequently reducing GSH synthesis by GCLC, an important enzyme in ferroptosis resistance [174]. Notably, exposure to non-toxic iron-doped cerium oxide nanoparticles induces ROS-independent Nrf2 activation through concurrent H3K4me3/H3K27me3 enrichment and MLL1 complex recruitment at the *NFE2L2* promoter. This suggests a new regulatory mechanism for ferroptosis absent in toxic nanoparticle exposure [175]. Given the potential therapeutic implications of modulating ferroptosis in oncology, understanding the direct effects of histone methylation on *NFE2L2* signaling is a crucial area for future research.

#### 5.2.3. Other Forms of Histone Modifications

Histone ubiquitination is a distinct type of PTM characterized by the covalent linkage of ubiquitin to histone lysine residues [176]. This process creates specific ubiquitination patterns. Ubiquitination is particularly versatile, as ubiquitin can exist in various polymerization states, with monoubiquitination typically associated with transcriptional activation, DNA repair, and trans-tail crosstalk. In contrast, polyubiquitination is linked to chromatin destabilization, transcriptional silencing, and maintenance of DNA methylation, and it also plays a role in DNA repair processes [177,178,179,180,181,182,183]. Notably, experimental models demonstrate UBR7-mediated monoubiquitination at H2B K120 within the *Keap1* promoter region in HCC. This modification suppresses tumorigenesis by modulating the Keap1/Nrf2/Bach1/HK2 signaling axis [184].

Although extensive research has outlined the role of chromatin histone ubiquitination and deubiquitination in disease development [185,186], its regulatory influence on *NFE2L2* expression and ferroptosis remains underexplored. Future studies could use chromatin immunoprecipitation sequencing to map the dynamic patterns of ubiquitination at the *NFE2L2* promoter during oxidative stress or ferroptotic challenges [187]. Additionally, employing genetic ablation or pharmacological inhibition of specific E3 ubiquitin ligases may help clarify how ubiquitination contributes to Nrf2-mediated ferroptosis.

### 5.3. Post-Transcriptional mRNA Modifications in NFE2L2 Expression and Ferroptosis

Post-transcriptional mRNA modifications refer to chemical changes that regulate RNA metabolism, including stability control, translational efficiency, and subcellular localization. Primary variants of these epitranscriptomic modifications include m^6^A, m^5^C, pseudouridylation (Ψ), 2′-O-methylation, adenosine-to-inosine editing, and 7-methylguanosine. These modifications are significantly relevant to various diseases, with substantial evidence linking their dysregulation to malignant transformation and cancer progression through altered redox homeostasis and stress response pathways.

#### 5.3.1. m^6^A Methylation of *NFE2L2* mRNA Regulates Ferroptosis

m^6^A is the most prevalent and well-characterized epitranscriptomic modification in eukaryotic mRNA. Its addition is catalyzed by a methyltransferase complex comprising METTL3, METTL14, and WTAP. Additionally, the RNA-binding protein HNRNPA2B1 performs analogous splicing regulatory functions to METTL3 through direct transcript interactions [188]. Demethylases such as FTO and ALKBH5 actively remove these modifications, while reader proteins, including YTHDF, YTHDC, and IGF2BP, recognize m^6^A marks to modulate RNA stability [189,190,191,192]. These readers regulate the degradation of m^6^A-modified transcripts via CCR4-NOT and ribonuclease P/MRP complexes while also influencing RNA secondary structures, splicing events, nuclear trafficking, and transcript longevity.

The preferential enrichment of m^6^A within pri-miRNA introns suggests critical roles in splicing regulation [192,193]. For instance, METTL3-mediated m^6^A deposition facilitates DGCR8-dependent pri-miRNA processing into mature miRNAs while stabilizing selected mRNAs [194]. In cisplatin-resistant HCC, METTL3 overexpression elevates m^6^A levels on *NFE2L2* mRNA transcripts, enhancing their stability and thereby suppressing ferroptosis [195]. Interestingly, neutrophil extracellular trap-activated METTL3 promotes m^6^A methylation of *HIF1A* mRNA. This recognition by IGF2BP2 reduces HIF-1α degradation, ultimately leading to decreased *GPX4* expression and increased sensitivity to ferroptosis [196]. Thus, the consequence of METTL3-mediated m6A modification is dependent on target mRNAs. The complexity of m^6^A regulatory networks is further exemplified by METTL4, exacerbating ferroptosis in sepsis-induced acute lung injury through Nrf2 hypermethylation [197].

Transcript stability modulation comprises another key m^6^A function. Hepatitis B virus elevates m^6^A levels on tumor suppressor *PTEN* mRNA, accelerating its decay to promote hepatocarcinogenesis [198]. Conversely, melatonin increases NEDD4 ubiquitin ligase stability through METTL3-mediated m^6^A modification, leading to SIRT6 ubiquitination and Nrf2/HO-1 pathway activation to inhibit ferroptosis [199].

FTO-mediated demethylation of *NFE2L2* mRNA reduces ferroptosis-driven cerebral ischemic injury, as indicated by lower expression levels of *FTO* and *NFE2L2* in MCAO/R rat models and OGD/R-treated SH-SY5Y cells. Complementary studies in epileptic rats reveal hippocampal neuron m^6^A hypermethylation, which coincides with a decrease in FTO and an increase in YTHDF2-mediated degradation of *NFE2L2* mRNA, impairing cellular viability. Interestingly, BPA-exposed TM3 cells demonstrate that YTHDF2 depletion enhances the stability of *NFE2L2* transcripts in an m^6^A-dependent manner [200,201]. Functional redundancy within YTHDF proteins and the gene-specific m^6^A effect underscore the necessity for mechanistic studies elucidating YTHDF2-Nrf2 interactions in ferroptosis.

The complex roles of m^6^A regulators in ferroptosis are exemplified by ALKBH5. Although it shares demethylase activity with FTO, ALKBH5 enhances ferroptosis sensitivity in hypopharyngeal squamous cell carcinoma by destabilizing transcripts [202]. Conversely, IGF2BP2 stabilizes *NFE2L2* mRNA, while IGF2BP3 overexpression helps maintain anti-ferroptotic transcripts (GPX4, SLC3A2, ACSL3, and FTH1) and confers sorafenib resistance in HCC [203,204]. These findings collectively illustrate that epigenetic regulation of Nrf2-mediated ferroptosis resistance constitutes a multifaceted network rather than a linear pathway.

#### 5.3.2. The m^5^C Methylation of *NFE2L2* mRNA

The m^5^C modification involves the enzymatic transfer of a methyl group from SAM to the fifth carbon position of cytosine bases within RNA molecules, producing m^5^C. This epi-transcriptomic modification plays a significant role in regulating RNA stability, translational efficiency, and cellular adaptation to stress [205]. Additionally, the m^5^C deposition is catalyzed by methyltransferases, including NSUN family proteins and DNMT homologs. Recognition of this modification is facilitated by reader proteins such as ALYREF, YBX1, and TET dioxygenases. Emerging evidence suggests that m^5^C is involved in remodeling the tumor microenvironment, immunotherapeutic responses, and cancer cell metabolic reprogramming. This is achieved through its effects on RNA localization, degradation rates, and structural stability [206,207].

Notably, m^5^C modifications demonstrate functional significance in ferroptosis resistance through Nrf2 pathway regulation. In NSCLC, *NSUN2* overexpression is strongly associated with advanced tumor grades and increased proliferation rates [208,209]. Mechanistic studies indicate that NSUN2-mediated hypermethylation of m5C stabilizes *NFE2L2* transcripts by facilitating their recognition by YBX1. This process upregulates ferroptosis-protective effectors, including GPX4 and FTH1, thereby conferring resistance to FINs. Despite these findings, the complex interplay between Nrf2-specific m^5^C methylation and ferroptotic susceptibility remains underexplored, with the current literature offering limited insights into this important regulatory mechanism.

## 6. Impact of PTMs of Nrf2 on Ferroptosis

PTMs are not typically classified as standard epigenetic modifications, as their primary function lies in modulating cellular activities at the protein level rather than through nucleic acid regulation. These biochemical modifications critically expand proteome functionality. Furthermore, these alterations include the covalent attachment of functional groups or proteins, proteolytic processing of protein subunits, and targeted protein degradation, serving as adaptive mechanisms to counteract oxidative stress and modulate cellular susceptibility to ferroptosis [210,211]. Recent studies underscore a significant interaction between Nrf2 PTMs and ferroptotic pathways, revealing novel regulatory nodes in redox homeostasis.

### 6.1. Restriction of the Nuclear Localization of the Nrf2 Functional Domain Affects Ferroptosis

DOX, a commonly used chemotherapeutic agent, exhibits dose-dependent cardiotoxicity, partly mediated through ferroptotic mechanisms [212,213]. In DOX-treated cardiomyocytes, protein arginine methyltransferase 4 interacts directly with the Neh4-6 domain of Nrf2, inducing arginine methylation that impedes nuclear translocation. This epigenetic modulation suppresses *GPX4* expression, thereby potentiating ferroptosis [214]. Conversely, Nrf2-mediated ferroptosis resistance may occur through enhanced promoter occupancy at AREs [215]. Notably, zinc finger MYND-type containing 8 (ZMYND8) recruits Nrf2 to antioxidant gene promoters in mammary epithelium. This creates a self-reinforcing regulatory circuit where Nrf2 overexpression leads to increased levels of ZMYND8, attenuating ROS accumulation and ferroptotic susceptibility. This reciprocal interaction sustains breast cancer stem cell (BCSC) stemness and tumorigenic capacity through redox adaptation [216]. Beyond histone acetylation-mediated regulation, direct Nrf2 acetylation modulates its ability to bind DNA. The CREB-binding protein, a HAT, catalyzes the lysine acetylation within Nrf2′s Neh1 domain, reducing its affinity for ARE binding and compromising the transcriptional activation of the cytoprotective gene [217].

### 6.2. Ubiquitination Modifications of Nrf2 Affect Ferroptosis

The Keap1-Cullin3 E3 ubiquitin ligase complex is a fundamental pathway for ubiquitination to cellular proteostasis. Beyond its well-characterized role in Nrf2 regulation, this complex mediates ubiquitination of the ER autophagy receptor Reticulon 1, thereby suppressing proteasomal degradation of BCL2 and p62. Genetic changes in Keap1 can lead to homeostatic imbalances, resulting in dysregulated apoptosis, autophagy, and inflammatory signaling cascades [218,219,220]. Under homeostatic conditions, Keap1 regulates constitutive Nrf2 degradation via the ubiquitin–proteasome system [221]. Oxidative stress induces structural changes in critical Cys residues within Keap1, allowing Nrf2 for nuclear translocation [215,222]. Within the nucleus, Nrf2 forms a heterodimer with sMaf proteins to identify AREs and electrophile response elements in target gene promoters, initiating transcription of cytoprotective antioxidant enzymes [90,108,221,223,224].

#### 6.2.1. Disruption of the Keap1/Nrf2 Binding Alleviates Ferroptosis

Evidence from OGD/R models of myocardial infarction in cardiomyocytes and macrophages reveals that paeoniflorin A covalently targets conserved Cys residues (Cys77 and Cys434) within Keap1. This interaction promotes structural destabilization of the Cul3-Keap1 ubiquitin ligase complex and Keap1/Nrf2 binding, ultimately activating the Nrf2 signaling pathway [222,225]. Complementary studies in DOX-induced ferroptotic H9C2 cardiomyocytes reveal that overexpression of FTO enhances the demethylation of *p21* mRNA, stabilizing Nrf2 and facilitating its nuclear translocation. Interestingly, p53 exhibits dual regulatory effects on Nrf2 via the p21 axis: Low p53 levels suppress *NFE2L2* expression, while elevated p53 concentrations promote its activity [226,227]. Pharmacological administration of bioactive compounds activates SIRT1-mediated p53 deacetylation, thereby enhancing Nrf2-driven redox homeostasis through ROS-scavenging antioxidant induction. This SIRT1/p53/Nrf2 regulatory axis demonstrates therapeutic potential in diabetic nephropathy pathogenesis [228]. Acetylation modifications competitively disrupt Keap1/Nrf2 interactions. Treatment of microglial cells with the histone deacetylase inhibitor Romidepsin results in increased Nrf2 acetylation levels, concurrently ameliorating inflammatory responses and ferroptosis [229].

Interestingly, increased levels of cytosolic Nrf2 and a reduction in Nrf2 nuclear localization along with an increased expression of KEAP1 in females are induced by a high-fat diet enriched with liquid fructose, while this phenotype was not observed in males [230]. In hyperglycemic conditions, supplementation of estrogen and/or estrogen receptors restores Nrf2, nNOSα, total nitrite, and nitrergic relaxation and further restores gastric neuromuscular function [231]. This finding indicates that sex hormones, especially female hormones, are critical for Nrf2 function.

#### 6.2.2. Impact of Keap1 Mutations on Nrf2 PTMs and Ferroptosis

Beyond oxidative stress, somatic mutations in Keap1 are a significant factor in the pathological activation of Nrf2. In patients with lung adenocarcinoma (LUAD), loss-of-function mutations in Keap1 lead to sustained upregulation of Nrf2, conferring therapeutic resistance to chemoradiation modalities [232,233,234]. Comprehensive methylation analyses of LUAD cohorts with Keap1 mutations reveal epigenetic dysregulation of several antioxidant-associated genes, including *GPX2*, *GCLC*, *TXNRD1*, *AKR1C1*/*AKR1C2*, *PGD*, *SRXN1*, and *ABCC2*. Notably, most of these methylation changes are characterized by hypomethylation events that correlate with transcriptional activation. Most affected genes are recognized as canonical targets of Nrf2 [235].

## 7. Perspectives and Future Directions

This review summarizes the current understanding of the critical role that Nrf2 plays in regulating ferroptosis and its epigenetic modulation. The findings establish Nrf2 as a key regulator of ferroptosis suppression by coordinating iron homeostasis, reducing oxidative stress, and managing lipid peroxidation dynamics. Nrf2 is involved in GSH biosynthesis, ROS scavenging, and intracellular free iron sequestration. Recent evidence emphasizes the significant regulatory influence of epigenetic mechanisms on Nrf2 expression and activity. These mechanisms include DNA methylation, histone modifications (acetylation and methylation), and mRNA modifications (m^6^A and m^5^C). Additionally, PTMs, such as ubiquitination and acetylation, refine Nrf2 nuclear translocation and transcriptional efficacy, thereby establishing a multi-layered regulatory hierarchy in ferroptotic control. These insights enhance our understanding of the mechanistic comprehension of Nrf2 and also identify potential therapeutic targets for diseases related to ferroptosis, including various types of cancer and neurodegenerative disorders.

Despite significant progress, critical knowledge gaps persist. First, the field needs a deeper understanding of how epigenetic regulation of *NFE2L2* varies by cell type and disease context. This can be achieved through advanced single-cell omics to resolve spatiotemporal modification dynamics. Second, rigorous correlation of *NFE2L2* epigenetic states with distinct pathological manifestations (carcinogenesis and I/R injury) could reveal the precise disease-modifying roles and inform targeted therapeutic development. Notably, the regulatory patterns observed in NSCLC, HCC, and BCSCs underscore the translational potential of this approach.

Future investigations should focus on three avenues: (1) the rational design of small-molecule compounds or epigenetic modulators to selectively manipulate *NFE2L2*/Nrf2 modifications; (2) a systematic exploration of natural compounds modulating Nrf2 activity as potential clinical therapeutics; (3) integrative multi-omics analyses (epigenomic, proteomic, and metabolomic) to construct comprehensive *NFE2L2*/Nrf2 regulatory networks in ferroptosis; and (4) other mechanisms (negative feedback, competitive inhibition, and time delays, among other complex interactions) regulating *NFE2L2* expression through epigenetic mechanisms. These interdisciplinary efforts will facilitate systems-level decoding of ferroptotic signaling, accelerating the translation of fundamental discoveries into diagnostic and therapeutic innovations for precision medicine.
